# The Maximal Pore Size of Hydrophobic Microporous Membranes Does Not Fully Characterize the Resistance to Plasma Breakthrough of Membrane Devices for Extracorporeal Blood Oxygenation

**DOI:** 10.3389/fbioe.2019.00461

**Published:** 2020-01-10

**Authors:** Gionata Fragomeni, Mara Terzini, Antonio Comite, Gerardo Catapano

**Affiliations:** ^1^Department of Medical and Surgical Sciences, Magna Graecia University, Catanzaro, Italy; ^2^Department of Mechanical and Aero-Space Engineering, Politecnico di Torino, Turin, Italy; ^3^Department of Chemistry and Industrial Chemistry, University of Genova, Genova, Italy; ^4^Department of Mechanical, Energy and Management Engineering, University of Calabria, Rende, Italy

**Keywords:** ECMO, membrane, plasma breakthrough, pore size, surfactants

## Abstract

Extracorporeal membrane oxygenation (ECMO) in blood-outside devices equipped with hydrophobic membranes has become routine treatment of respiratory or cardiac failure. In spite of membrane hydrophobicity, significant amounts of plasma water may form in the gas compartment during treatment, an event termed plasma water breakthrough. When this occurs, plasma water occludes some gas pathways and ultimately cripples the oxygenator gas exchange capacity requiring its substitution. This causes patient hemodilution and increases the activation of the patient's immune system. On these grounds, the resistance to plasma water breakthrough is regarded as an important feature of ECMO devices. Many possible events may explain the occurrence of plasma breakthrough. In spite of this, the resistance to plasma breakthrough of ECMO devices is commercially characterized only with respect to the membrane maximal pore size, evaluated by the bubble pressure method or by SEM analysis of membrane surfaces. The discrepancy between the complexity of the events causing plasma breakthrough in ECMO devices (hence determining their resistance to plasma breakthrough), and that claimed commercially has caused legal suits on the occasion of the purchase of large stocks of ECMO devices by large hospitals or regional institutions. The main aim of this study was to identify some factors that contribute to determining the resistance to plasma breakthrough of ECMO devices, as a means to minimize litigations triggered by an improper definition of the requirements of a clinically efficient ECMO device. The results obtained show that: membrane resistance to breakthrough should be related to the size of the pores inside the membrane wall rather than at its surface; membranes with similar nominal maximal pore size may exhibit pores with significantly different size distribution; membrane pore size distribution rather than the maximal pore size determines membrane resistance to breakthrough; the presence of surfactants in the patient's blood (e.g., lipids, alcohol, etc.) may significantly modify the intrinsic membrane resistance to breakthrough, more so the higher the surfactant concentration. We conclude that the requirements of ECMO devices in terms of resistance to plasma breakthrough ought to account for all these factors and not rely only on membrane maximal pore size.

## Introduction

Extracorporeal membrane oxygenation (ECMO) has become routine treatment of respiratory or cardiac failure patients, and has been proven effective for both adults and newborns (Bartlett et al., 2000; Betit, 2018). ECMO devices were first introduced in the ‘70s as an alternative to mechanical respirators and bubble oxygenators to substitute for the gas exchange function of the natural lung in the treatment of Acute Respiratory Failure (ARF) and in open heart surgery (Presenti et al., 1988). Since then, the use of membrane devices for blood oxygenation and CO_2_ removal (ECCO_2_R) in extra-corporeal circulation has reduced the mortality associated with ARF and the post-operative complications caused by mechanical respirators and bubble oxygenators. Regardless of the connection scheme to the blood circulation of the host, in ECMO and ECCO_2_R the membrane gas exchanger is required to operate without loss of gas transfer capacity from about 8 h up to 14 days, at least (Gaylor, 1988; Montoya et al., 1992; Shimono et al., 1996, 1997). Initially, ECMOs were mainly using homogeneous, dense silicone membranes as barriers between the blood and the gases. The use of microporous hollow fibers made of hydrophobic polyolefins (e.g., polypropylene, PP, and polyethylene) has permitted to enhance the effectiveness of gas exchange of ECMO devices. In fact, the polymer hydrophobicity prevents plasma water from entering the membrane pores and gases may freely and quickly diffuse through the stagnant gas trapped in the pores. As a result, oxygen transfer to blood through microporous membranes is limited only by the mass transport resistance of the stagnant layer of plasma at the blood-membrane interface, membrane resistance to transport being negligible (Gaylor, 1988). This has prompted the development of new ECMO designs where blood flows in patterns that promote mixing and minimize the resistance to oxygen transport of the stagnant blood layer adhering on the membrane. The Extra-Luminal Flow (ELF) concept is one of such new designs in which the blood flows outside hollow fiber membranes arranged in cross-wound mats whilst a gas, rich in oxygen and poor in carbon dioxide, flows inside the membrane lumen counter–/ or cross-currently (Catapano et al., 1992). Its efficiency significantly reduces the membrane surface area required to bring the patient blood to a given oxygen saturation level and has made it the reference design for clinical ECMO devices (Catapano et al., 1992, 2001).

In spite of the membrane hydrophobicity, the presence of significant amounts of blood plasma water in the membrane lumina during treatment has been occasionally reported in short-term ECMO and is a known complication of prolonged ECMO (Meyns et al., 2005). Such phenomenon is often referred to as “membrane wetting,” “plasma leakage,” or “plasma breakthrough,” the last one in analogy to solute outbreak in devices for liquid chromatography. When this occurs, blood plasma occludes some of the gas pathways, increases the resistance to gas transport in the gas compartment, and ultimately decreases or cripples the oxygenator gas exchange capacity. This requires substitution of the oxygenator, which causes patient hemodilution and increases the activation of the patient's immune system for the contact with additional foreign surfaces (Mottaghy and Hahn, 1981; Montoya et al., 1992). On these grounds, the use of ECMO devices equipped with microporous, hydrophobic membranes is not recommended for long-term respiratory support and resistance to plasma breakthrough is often regarded as an important feature of ECMO devices.

Many possible causes have been proposed to explain the occurrence of plasma breakthrough (Mottaghy and Hahn, 1981; Lund et al., 1998; Eash et al., 2004; Gill et al., 2015), from the presence of defects (e.g., pinholes) in the membrane wall, to water vapor condensation (Mottaghy et al., 1989) in the gas compartment, to excessively high transmembrane pressures (Tamari et al., 1991), to the adsorption of blood-borne surfactants on membrane pore wall which change its hydrophobicity (Kida et al., 2018), to quote but a few. In spite of this, the resistance to plasma breakthrough of ECMO membranes is commercially characterized with respect to one parameter only, the maximal pore size, which is generally evaluated with the bubble pressure method (Hernández et al., 1996), and occasionally estimated by scanning electron microscopy (SEM) analysis of the membrane surfaces.

The discrepancy between the complexity of the events causing plasma breakthrough in ECMO devices (hence determining their resistance to plasma breakthrough), and that claimed commercially has spurred controversy, and caused litigations and legal suits on the occasion of the purchase of large stocks of ECMO devices by large hospitals or regional institutions with significant costs for society at large. In this paper, we provide evidence of some factors that contribute to determining the plasma resistance of ECMO devices, as a means to minimize litigations triggered by an improper definition of the requirements that a resistant ECMO device has to meet. In particular, we provide evidence of the fact that membrane resistance to breakthrough should be related to the size of the pores inside the membrane wall rather than at its surface, that membranes with a nominal maximal pore size indeed exhibit pores with a size distribution that may significantly differ as an effect of small variations of the production parameters, and so its resistance to breakthrough, that the presence of surfactants in the patient's blood (e.g., lipids, alcohol, etc.) may significantly modify the intrinsic membrane resistance to breakthrough, more so the higher the surfactant concentration.

## Materials and Methods

### Membranes and Membrane Modules

In this study, microporous polypropylene membranes were used throughout that were prepared in small amounts by thermal induced phase separation (TIPS) with a laboratory spinning apparatus. They were kindly provided for this study by Membrana GmbH (Wuppertal, Germany) (now 3M™ Membrana™). The reference composition of the polymer dope and the extraction liquid, and the operating conditions were those used for the preparation of commercial microporous polypropylene membranes with 0.1 μm nominal maximal pore size, 40% porosity, 280 μm inner diameter, 50 μm wall thickness. Membranes with different pore structures were prepared by slightly changing some of the preparation parameters (e.g., polymer content, solvent, and extracting liquid composition) to simulate fluctuations potentially occurring during production in case of faulty quality control. Hereinafter, these membranes shall be referred to as Type I to VI. The maximal pore size of the investigated membranes was characterized by bubble point measurements and was consistently lower than 0.13 μm. To test their resistance to water breakthrough in the presence of surfactants, membranes of a given batch were assembled in laboratory-scale modules of about 0.167 m^2^ membrane surface area in a shell-and-tube configuration, their ends were potted with glue, and the membrane length protruding out of the cylindrical housing was cut with a sharp blade. This study was intended to guide membrane makers to develop membranes resistant to plasma breakthrough. For this reason, possible correlations between membrane pore structure and resistance to breakthrough were sought only for those membranes that exhibited a better resistance to breakthrough in preliminary experiments (i.e., Type I-IV). In such a case, possible time changes of membrane properties during the spinning procedure were minimized by using for testing only membranes spun in very short time intervals.

### Membrane Pore Characterization

Pore morphology and size of some membranes was characterized both at the outer membrane surfaces and inside the membrane wall by scanning electron microscopy (SEM) and by liquid porosimetry, as described below.

#### Scanning Electron Microscopy Analysis

Scanning electron micrographs were taken from different areas of membrane samples with a Stereoscan S-440 (Leica-Cambridge Leo, Wetzlar, Germany) SEM operated at accelerating voltages of 10 and 20 kV. Membrane samples were prepared for SEM by drying, breakage in liquid nitrogen, fixation to a SEM spin stub with a conductive adhesive, and sputter coated with a 20 nm layer of gold. Micrographs were taken of both membrane outer and inner surfaces and of membrane cross-sections.

#### Liquid Porosimetry Analysis

The distribution of pore size in the membrane wall and the relative hydraulic permeability (i.e., the fractional permeating flow rate) associated with pores in a given size range was characterized by liquid-liquid displacement porosimetry (LLDP) with a proprietary automated porosimeter (Calvo et al., 2004). Measurements were performed with an isobutanol–water pair, using isobutanol for wetting and water to displace it at increasing operating pressures, at 20°C. Membrane samples from any given batch were immersed in the LLDP wetting phase under vacuum to ensure complete membrane wetting. The pore radius, *r*_*p*_, corresponding to the measured volumetric flow rate of displaced water at any given operating pressure was estimated from the current pressure value, and for cylindrical straight pores, according to Laplace Equation

(1) $$\begin{array}{l} \Delta \text{P}=-\;\frac{2\;\gamma \;cos\theta }{r_{p}} \end{array}$$

Equation (1) is the force balance inside a cylindrical straight through-pore in a membrane, and relates the pressure, ΔP, at which a liquid breaks through the pore of size *r*_*p*_, for a surface tension of the liquid–air- membrane interface γ, and a membrane contact angle with water θ. In the analysis of the LLDP curves, γ was taken as the surface tension of the water–isobutanol-membrane interface, and θ = 0 for complete wetting.

### Effect of Surfactants on Water Breakthrough

**Figure 1:**
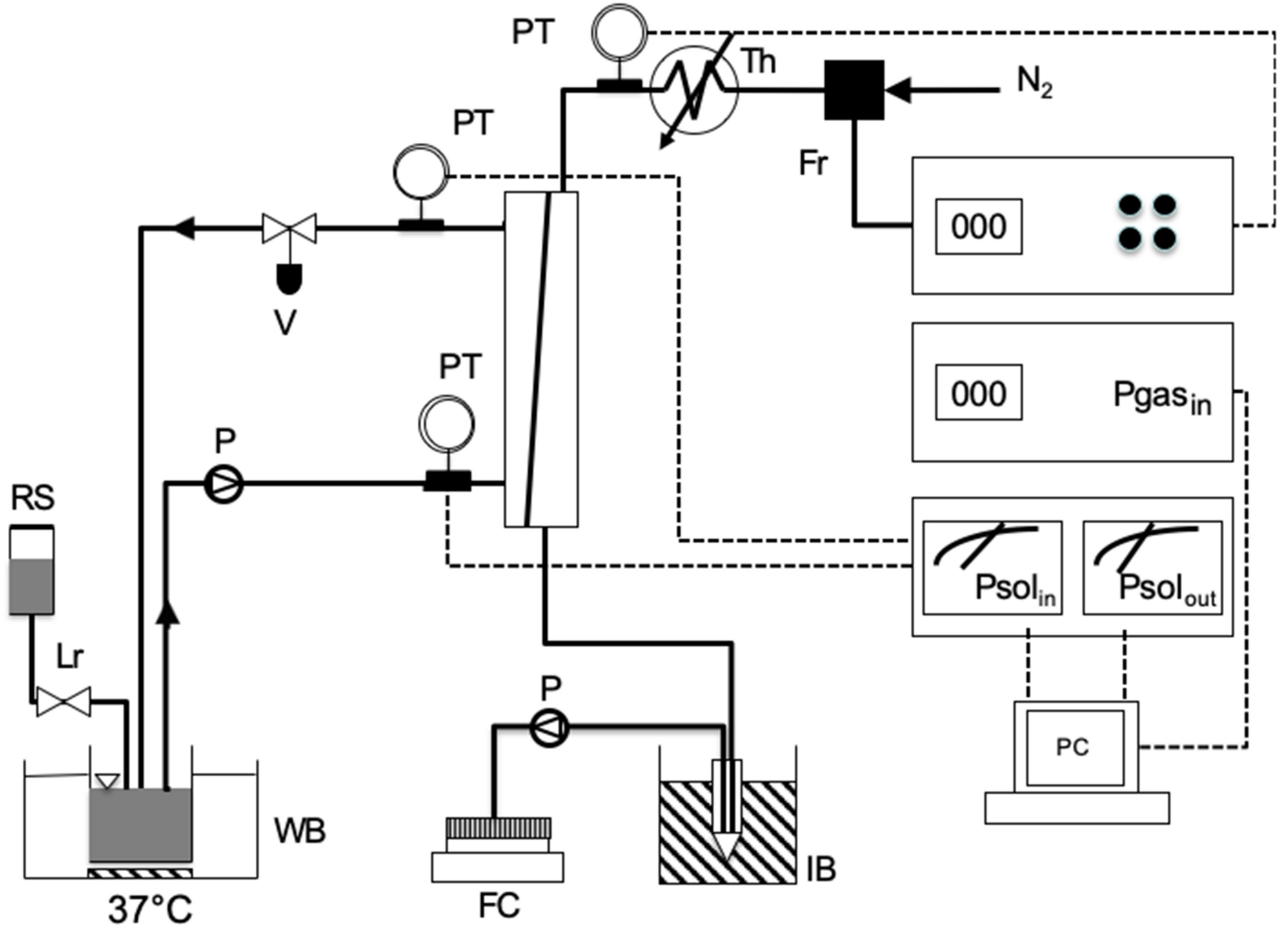
Scheme of the experimental apparatus used for testing the resistance to water breakthrough of modules equipped with the investigated membranes: FC, fraction collector; FR, flow controller of gas stream; IB, ice bucket; LR, level controller; P, pump; PC, personal computer; PT, pressure transducer; RS, reservoir with solution; TH, thermostat for gas heating; WB, thermostated water bath.

The experimental apparatus used to test membrane resistance to water breakthrough in the presence of surfactants is schematically shown in Figure 1. Before any experiment, a module was rinsed by flowing 1 L of 0.9% sodium cloride saline solution in the module shell in single-pass mode at 240 mL/min, at room temperature. Then, 200 ml of saline solution, previously warmed to 37°C, was recirculated in closed loop trough the module at the same flow rate for 10 min. While rinsing the module, gaseous nitrogen warmed at 38°C was kept flowing at 3 L/h in the membrane lumen counter-currently with respect to saline.

Membrane resistance to water breakthrough was characterized with respect to saline solutions containing egg yolk L-α phosphatidylcholine (Type XV-E, Sigma, Milan, Italy) at concentrations ranging from 0.37 to 12 g/L. Resistance to water breakthrough of membranes Type I to IV was characterized with solutions containing 3.1 g/L phosphatidylcholine. In the experiments, 200 mL of test solution kept at 37°C was recirculated in closed loop at a flow rate of 240 mL/min between module shell and the reservoir hosting the solution, while the level of the test solution in the reservoir was continuously controlled with a level-controller to avoid the solution concentration by evaporation. Little liquid losses were replenished with saline. Gaseous nitrogen, warmed at 38°C, was kept flowing counter-currently in the membrane lumen at 3 L/h. The pressure at the module sections at which the test liquid solution would enter or leave the module was continuously monitored, so was the inlet gas pressure. Water vapor in the gas stream leaving the module was continuously condensed in an ice-cooled tube and the condensate was continuously sent with a pump to a fraction collector for further condensate analysis. The water flow rate leaving the module through the gas side, *Q*_*w*_, was estimated in terms of the mass of condensate collected in 1 h. A patent membrane module was used for each experiment. Experiments to assess the effect of phosphatidylcholine concentration on the water breakthrough time were performed on four modules equipped with membranes Type V or VI at each concentration. When one or a few membranes in a module proved faulty (e.g., exhibiting pinholes) the experiment was discarded. The number of successful experiments modules tested under any given condition was not deemed high enough to perform a statistical analysis.

**Figure 2:**
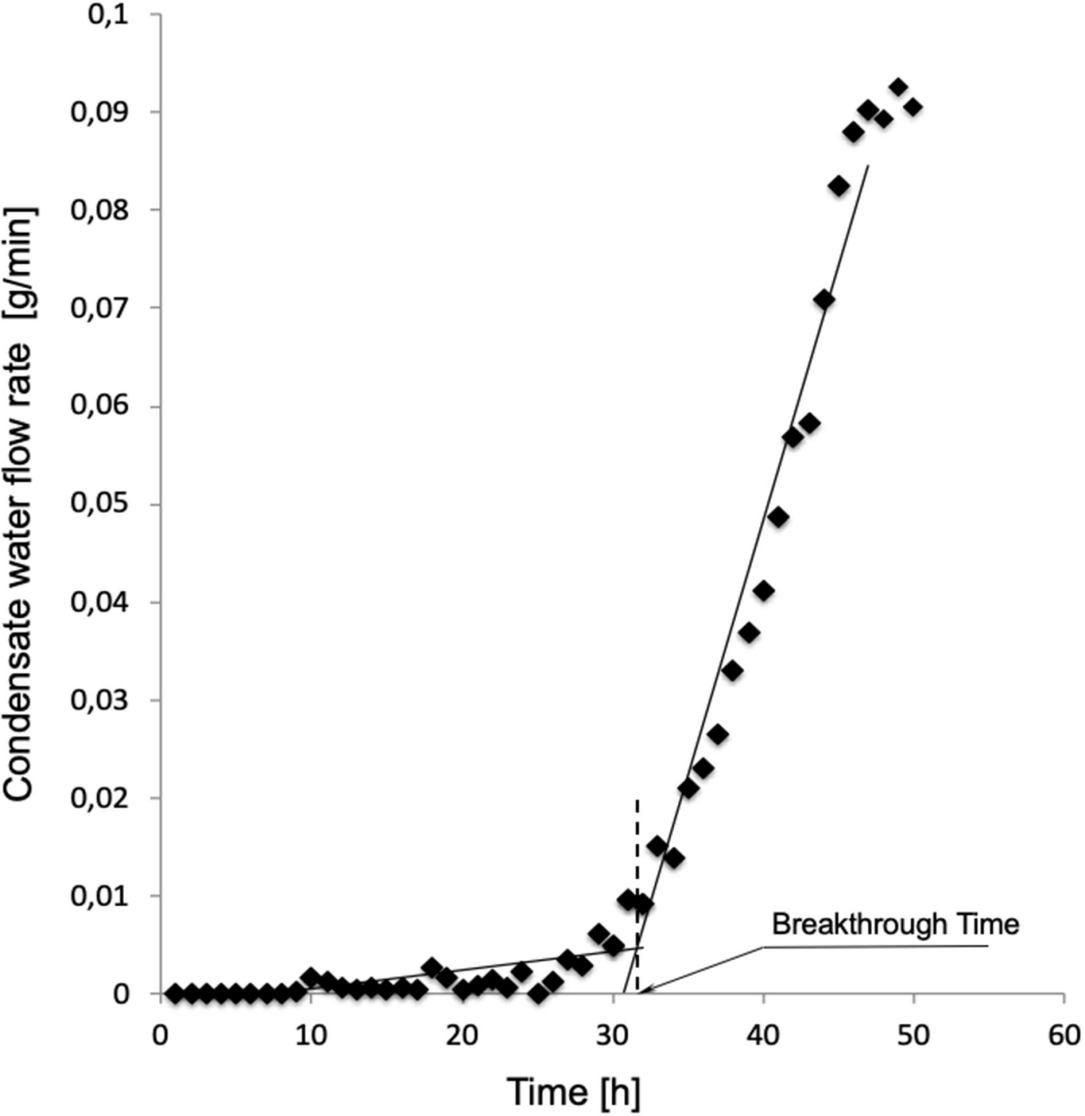
Typical time variation of the water flow rate leaving the module through the gas side of modules equipped with membranes Type I. Experiment performed as described in Materials and Methods with solutions containing 3.1 g/L phosphatidylcholine.

Figure 2 shows a typical water breakthrough curve expressed in terms of time variation of Q_w_. The time to water breakthrough, BT, for any membrane investigated in this study was estimated from experimental Q_w_ vs. t curves as the time coordinate of the intercept between the linear parts of each Qw vs. t curve at the beginning and toward the end of the experiment. Phospholipid penetration into the membrane pores and adsorption on their surface was investigated by assessing the presence of phosphatidylcholine by SEM elemental analysis of membrane cross-sections at the end of the experiments.

## Results

### Membrane Pore Characterization

**Figure 3:**
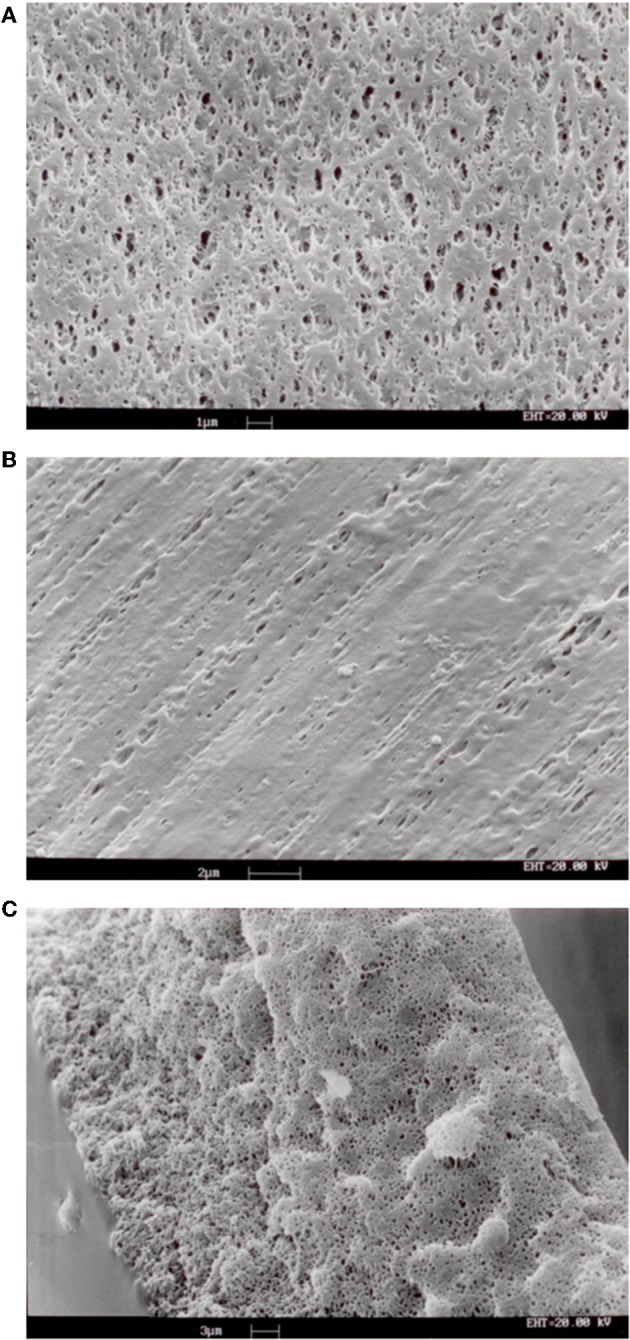
SEM images of samples of membranes Type I: **(A)** inner surface; **(B)** outer surface; **(C)** cross-section of membrane wall.

SEM analysis of the investigated membranes typically showed that pore density and size was rather different at the two membrane surfaces and along the membrane thickness. Pores on the surfaces were bordered with stretches of polymer, of varying surface area, and were generally elongated. As shown in Figures 3A,B, pore density was lower at the outer membrane surface, that contacting the blood in ELF ECMO device, than the inner membrane surface. Figure 3C shows that, inside the membrane wall, pores appeared as rather spherical voids (different than the finger-like voids typical of hemodialysis membranes) and their density decreased from the outer to the inner membrane surface. Only a fraction of pores appeared to be through pores, definitely no cylindrical and straight pore was to be seen, opposite to that often assumed in many theoretical models.

**Figure 4:**
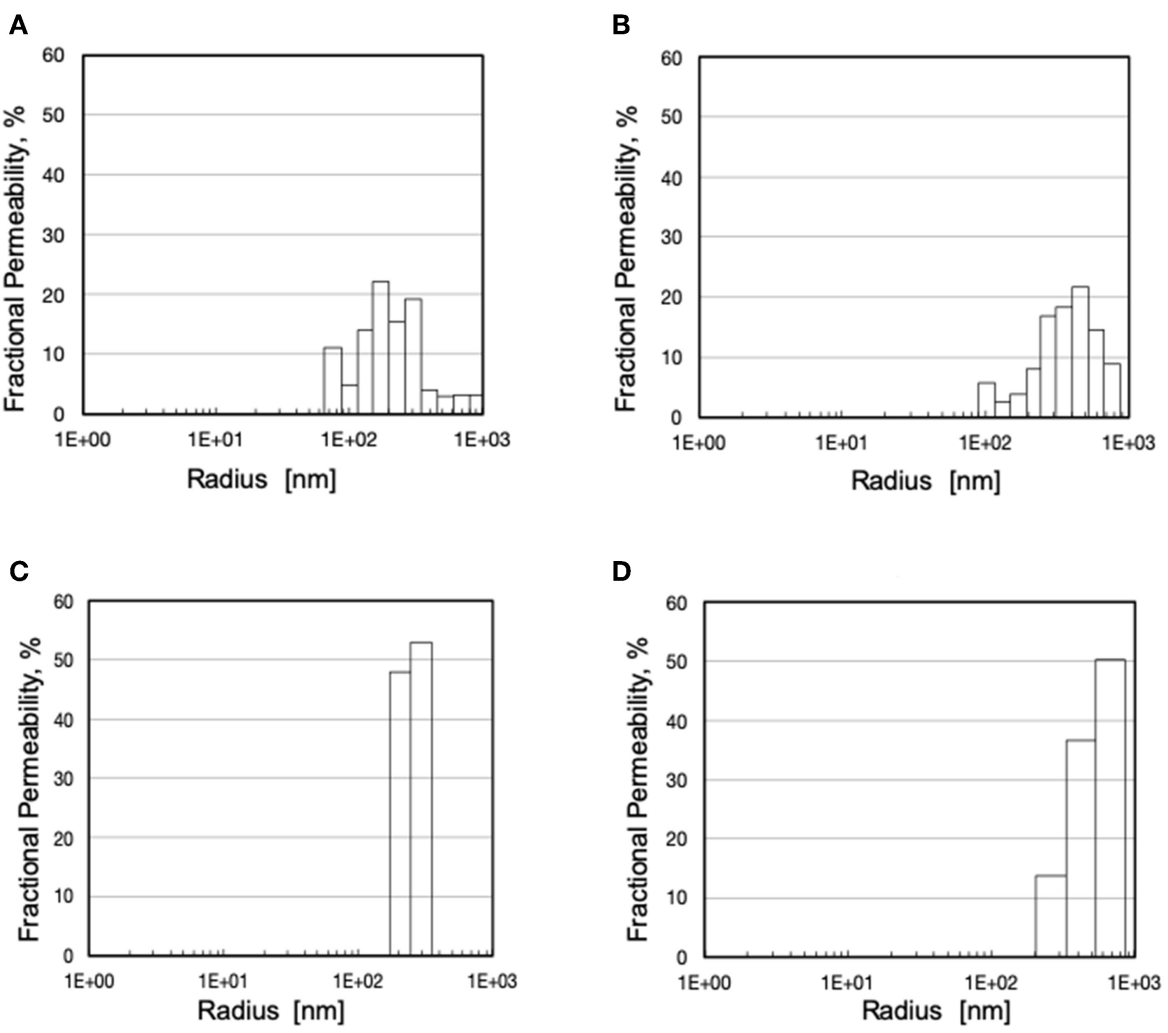
Pore size distribution and fractional hydraulic permeability of various membranes: **(A)** Type I; **(B)** Type II; **(C)** Type III; **(D)** Type IV.

The analysis based on liquid-liquid displacement porosimetry permitted to characterize the effective transport features of the pores inside the wall of the investigated membranes. As shown in Figure 4, small variations of the preparation parameters yielded membranes with significantly different pore size distributions. Albeit to varying extent, all membranes exhibited pores greater than the nominal maximal pore, and only a few exhibited pores smaller than that. The pore size distribution was also broader than expected, for some of them. Figure 4C shows that only the Type III membranes exhibited a very narrow pore size distribution with mean pore size close to the nominal maximal pore size.

### Effect of Surfactants on Water Breakthrough

Figure 2 shows a typical water breakthrough curve in terms of time variation of the mass flow rate of water leaving the module through the gas side. At the beginning of the test, water is transported across the membrane wall as a vapor at a rather constant and very low flow rate, generally well below 10^−3^ g/min. After water breakthrough occurs, water is mainly transported across the membrane wall as a liquid at a rate that continuously increases in time. Water breakthrough was evidenced by the corresponding pressure increase (up to about 4 times the initial pressure) of the gas stream entering the membrane module (hence membrane lumina). The pattern of time increase of the water flow rate after breakthrough varied with the membrane type and its pore size distribution. Transition from the former to the latter regime occurred in a time interval that varied with the membrane type and its pore size distribution. In some cases, elemental analysis evidenced the presence of phosphatidylcholine adsorbed on the pore surface inside the membrane wall caused by the flow of the liquid solution across the pores after breakthrough occurred.

**Figure 5:**
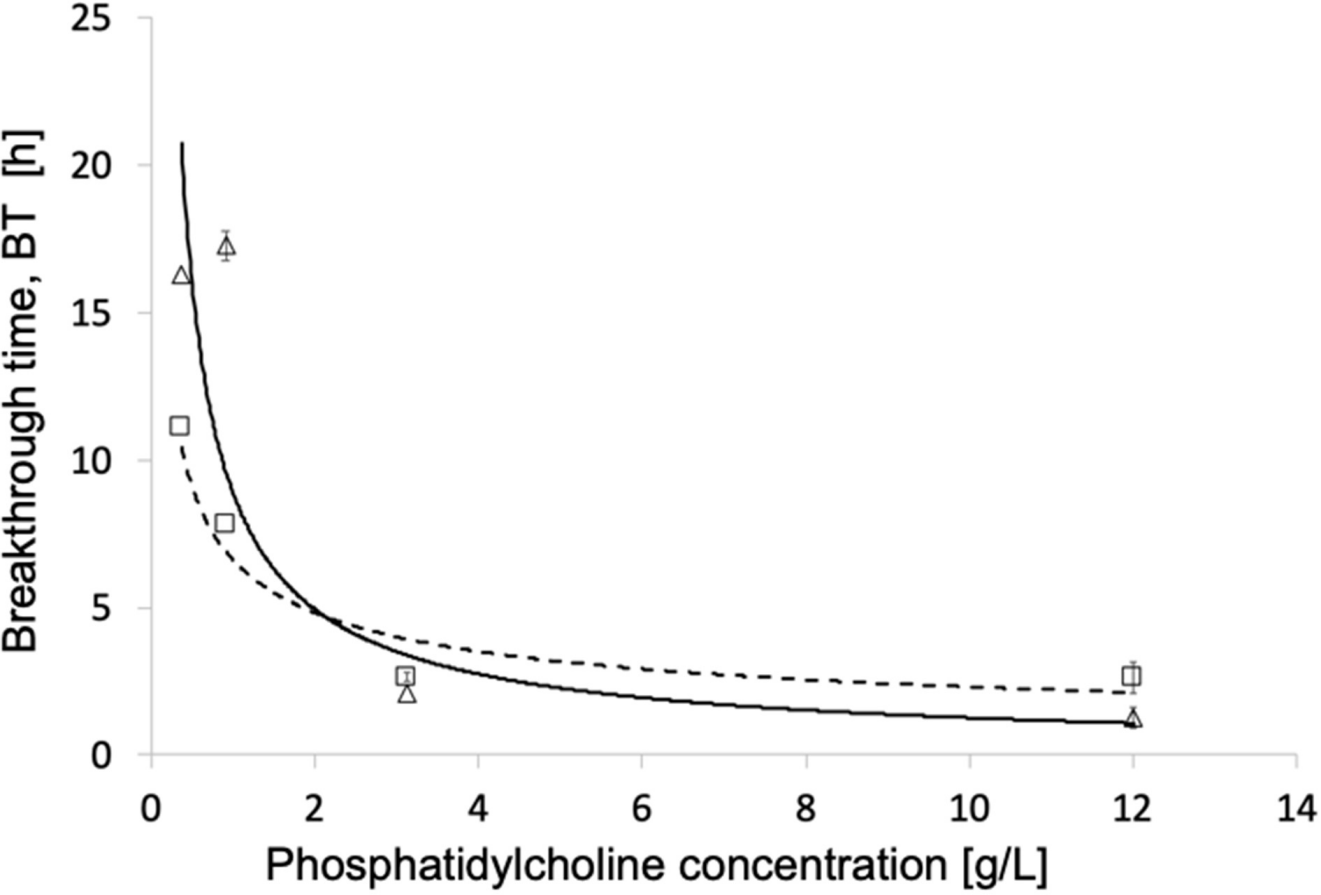
Breakthrough time for different phosphatidylcholine concentrations of modules equipped with different membranes: (Δ, **- - -**) Type V; (□, ^**____**^) Type VI. Other operating conditions as in Materials and Methods.

In the presence of phosphatidylcholine concentrations slightly higher than in plasma, all investigated membranes exhibited water breakthrough times well in excess of that required for their use in ECMOs for open heart surgery. The experimental breakthrough times varied with the membrane pore size distribution. Table 1 shows that the breakthrough times are better correlated to the membrane mean pore size than the maximal pore size, and significantly decreased with increasing mean pore size. Figure 5 shows that the water breakthrough time decreased exponentially when the membranes were exposed to solutions containing increasing concentrations of phosphatidylcholine. Figure 5 shows that the breakthrough time was not significantly affected by the membrane properties when membranes were exposed to high phosphatidylcholine concentrations. Membrane properties significantly affected the breakthrough time when membranes were exposed to lower phosphatidylcholine concentrations, in the range of the values typically found in human plasma.

**Table 1:** Breakthrough time, BT, of modules equipped with the investigated membranes as a function of their mean, R_mean_, minimal, R_min_, and maximal, R_max_, pore size.

**Membrane type**	**Membrane features**			
	**R_**mean**_ [nm]**	**R_**min**_ [nm]**	**R_**max**_ [nm]**	**BT [h]**
I	257	66	1,070	30.7
II	404	90	873	25.0
III	240	181	369	>43.0
IV	560	213	873	15.6

*Experiments performed as described in Materials and Methods with solutions containing 3.1 g/L phosphatidylcholine*.

## Discussion

The efficiency of microporous hydrophobic membranes in transporting gases to/from the blood in ECMO is mainly due to the fact that gases quickly diffuse across the column of stagnant gas present in the membrane pores, while polymer hydrophobicity prevents plasma water from crossing the membrane. On purely theoretical grounds, the absence of a dense barrier between the gas and the blood stream could lead to the formation of micro-emboli in the blood that could expose ECMO patients to serious hazards. As it can be estimated from Equation (1), for polyolefin membranes with maximal pore size a tenth of a micron large, microbubble formation may occur only when the oxygenator is operated at high gas pressures (e.g., when gas flow rate is increased to recover some gas exchange capacity lost for plasma leakage) (Gill et al., 2015) or when pinholes are present in the membrane wall (i.e., with faulty membranes). In this study, micro-emboli formation was not investigated because experiments with faulty membranes were discarded and gas flow rate was not increased when plasma leakage occurred because the study was not focused on the characterization of the oxygenator gas exchange capacity.

Equation (1) shows that pore size, but also the interfacial surface tension, γ, and the membrane contact angle, θ, contribute to determining the barrier properties of microporous membranes. It should also be considered that synthetic membranes prepared by phase inversion generally exhibit pores the size of which may be broadly distributed, depending on the actual membrane preparation procedure. In spite of this, the barrier properties of microporous membranes for ECMO are generally characterized only in terms of their maximal pore size estimated by bubble point measurements (Tylkowski and Tsibranska, 2015), or by SEM analysis of the membrane surfaces, as is often the case in commercial advertisements. Such an oversimplified characterization of the resistance to plasma breakthrough of ECMO devices has often been used in the definition of the requirements that effective and safe ECMO devices have to meet to qualify for purchasing by large hospitals or regional institutions. The discrepancy between the many factors causing plasma breakthrough in ECMO devices and the simplicity of that required commercially has often led to legal disputes in a court of law. This study was designed to evidence the limits of using only the membrane maximal pore size to characterize the resistance to plasma breakthrough of ECMO devices, and to bring experimental evidence of the extent to which the whole membrane pore size distribution and the conditions under which an ECMO device is operated determine the occurrence of water breakthrough. In the clinics, the occurrence of plasma breakthrough is signaled by a decrease of the ECMO gas exchange capacity (e.g., Gill et al., 2015), an effectiveness parameter strongly dependent on the actual fluid dynamics of the ECMO device (Catapano et al., 2001). It is worthwhile noting that the methods used for this study permitted to characterize the occurrence of plasma breakthrough independent of measurements of the gas exchange across the membrane wall, hence of the actual ECMO module design.

The SEM analysis in Figure 3 clearly shows that pores at the membrane surfaces do not fully characterize membrane resistance to breakthrough. In fact, not all surface pores are through-pores, nor does their geometry and size at the membrane surface always resemble those inside the membrane wall. In agreement with Hernández et al. (1998), it is the pores inside the membrane wall that determine the membrane transport properties, unless a selective layer (i.e., a skin) is present at one of the membrane surfaces. Equation (1) permits to estimate the pressure at which water breaks through a membrane that features through-pores all of which are cylindrical, with uniform size, and have the same radius, *r*_*p*_. In spite of the fact that the maximal pore size of the investigated membranes was lower than 0.13 μm, the LLDP analysis reported in Figure 4 showed that they generally featured a relatively broad pore size distribution, and exhibited a significant fraction of pores larger than their maximal pore size. Extension of Equation (1) to membranes featuring pores of varying size suggests that the membrane resistance to breakthrough ought to reflect the actual membrane pore size distribution.

To investigate whether this is indeed the case, the resistance to water breakthrough of the investigated membranes was assessed in terms of the time it would take for water to break through the membranes when they are continuously challenged with a solution containing a surfactant. This would simulate that happening during an ECMO treatment. In fact, the blood contains many surface-active species at relatively high concentrations (e.g., proteins and lipoproteins), more so under pathological conditions (e.g., in case of elevated serum lipoproteins and/or hypercholesterolemia). Exogenous surface-active species may also be present in the patient's blood for his/her personal habits (e.g., alcohols), or because they are given to the patient in large amounts to treat cardio-pulmonary diseases (e.g., lipophilic drugs). The presence of surfactants in the blood, or the test solution, has two important consequences. Firstly, they significantly decrease the blood surface tension with respect to water (Montoya et al., 1992). Secondly, they may adsorb on the hydrophobic polymer of which ECMO microporous membranes are made and decrease their contact angle, θ (i.e., make them more hydrophilic). In fact, such surface-active species are all amphiphilic (i.e., they possess both hydrophilic and hydrophobic character). When they come in contact with a hydrophobic polymer, they adsorb on them via their apolar domains to an extent depending on their blood concentration and affinity to the polymer, and expose their polar domains toward water. As more surfactant molecules adsorb on the polymer surface, they may end up forming a hydrophilic layer on top of the hydrophobic polymer. This decreases the polymer contact angle and permits plasma water to leak through the pores (Ishihara et al., 1993; Philp et al., 1994). In this study, a phospholipid was used as surface-active species because phospholipids are ubiquitous in the body. They are present in plasma at concentrations ranging from 1.58 to 2.84 g/L. The phospholipid concentrations used for this study were chosen to encompass their physiological range and to simulate pathological conditions, too. Consistent with that expected, Figure 2 and the presence of phospholipids adsorbed on the membrane wall show that after water breakthrough occurs water is mainly transported across the membrane wall as a liquid at a rate that continuously increases in time as an effect of phospholipid adsorption which causes wetting of pores of decreasing size.

Comparison of the water breakthrough times at phospholipid concentrations close to the upper physiological value (Table 1) and the pore size distributions (Figure 4) exhibited by the investigated membranes shows that, indeed, the entire membrane pore size distribution strongly influences the membrane resistance to breakthrough. Membranes with a significant fraction of large pores (i.e., Type II and IV) exhibited a short time to breakthrough (and a poor resistance to water breakthrough), the former shorter than the latter for the distribution slanted toward pores close to the micron in size. Membranes with a narrow pore size distribution exhibited a much longer time to breakthrough (and a better resistance to water breakthrough) than those with a broad pore size distribution (i.e., Type III vs. Type I), in spite of the rather close mean pore sizes (i.e., 240 vs. 257 nm, respectively). Interestingly, the breakthrough time correlated better with the mean pore size than the maximal pore size, possibly because the former accounts better for the entire pore size distribution of the investigated membranes.

Figure 5 shows that the exposure to increasingly high phospholipid concentrations, exponentially decreases the membrane water breakthrough time and minimizes the effects of the different membrane pore structures. This is possibly due to the fact the overwhelming presence of surface-active species in the test solution quickly decreases the membrane contact angle of all pore surfaces, and makes all of them wettable about at the same time irrespective of differences in size. This result is consistent with the premature breakthrough of plasma water across microporous membranes for ECMO reported in the cardiac surgery of alcoholics (Tylkowski and Tsibranska, 2015). It is worthwhile noting that at concentrations of surface-active species in the test solution ≤ 1 g/L the actual membrane structure significantly affects the membrane breakthrough time, and may make it increase by up to a factor two.

## Conclusions

In this paper we provide experimental evidence that the membrane maximal pore size only (estimated by the bubble point method or by SEM analysis of membrane surface) does not fully characterize the resistance to plasma water breakthrough of ECMO devices equipped with hydrophobic microporous membranes. Different phenomena may occur at the same time and may act cooperatively to initiate or favor plasma breakthrough. The experimental characterization shows that membrane resistance to breakthrough is better related to the size of pores inside the membrane wall rather than at its surface; that membranes with similar nominal maximal pore size may exhibit pores with significantly different size distribution; that membrane pore size distribution rather than the maximal pore size determines the membrane resistance to water breakthrough; that the presence of endogenous (e.g., excess lipoproteins or cholesterol) or exogenous (e.g., alcohol or drugs) surface-active species in the patient's blood may strongly modify membrane hydrophobic character and its intrinsic resistance to breakthrough, more so the higher the species concentration.

The results of this study suggest that litigations and legal suits might be minimized, in the first place, by avoiding that administrative offices issue calls for purchase in which improper definitions, based only on their membrane maximal pore size, are used of the requirements that clinically effective ECMO devices have to meet to be capable of resisting plasma breakthrough during prolonged treatments. In the second place, policies should be put in place to let authorized laboratories only test commercially available ECMO devices and membranes under standardized operating conditions so as to define the conditions of use under which commercially available ECMO devices deliver reproducible gas exchange performance and are safe in all possible clinical use (i.e., short–/ or long-term).

## Data Availability Statement

The datasets generated for this study are available on request to the corresponding author.

## Author Contributions

GC contributed conception of the study. AC performed and analyzed the experiments to characterize membrane structure. GF and MT performed the breakthrough experiments. GC, GF, and MT analyzed the breakthrough experiments. GF wrote the first draft of the manuscript. AC, GC, and MT wrote sections of the manuscript. All authors contributed design of the study, contributed to manuscript revision, read, and approved the submitted version.

### Conflict of Interest

The authors declare that the research was conducted in the absence of any commercial or financial relationships that could be construed as a potential conflict of interest.
